# Cholera at Jamaica

**Published:** 1851-03

**Authors:** 


### ARTICLE XII.

### *Cholera at Jamaca.*

The city of Kingston is said to have lost not fewer than 5,000 inhabitants by the cholera. The scourge was beginning to slacken at the last accounts. The Kingston Journal says: It has appeared at Radner, a property 3,000 feet above the level of the sea, and the finest climate known on the face of creation; and it has touched similar altitudes in the parishes of Port Royal and St. Andrew. It has been frightfully malignant at Midleton coffee plantation, and has manifested itself at Charlottenburg, Chester Vale, Newton, and other properties, all situated at an altitude that has hitherto defied febrile diseases.—*Scalpel.*

